# Evaluation of androgen receptor and androgen receptor splice-variant 7 in bladder cancer; a novel approach into an ancient topic

**DOI:** 10.1007/s00345-024-05166-z

**Published:** 2024-07-31

**Authors:** Ege Alper Sarıkaya, Peyda Korhan, Canet Incir, Alperen H. Yıldız, Dogan M. Deger, Selçuk M. Özer, Yesim Tuncok, Gul Ergor, Yasemin Ö. Islakoğlu, Volkan Sen, Ozan Bozkurt, Neşe Atabey, Adil A. Esen

**Affiliations:** 1 Dokuz Eylul University Hospital Urology Department, Edirne, Turkey; 2 Izmir Biomedicine and Genome Center, Balcova, Izmir 35340 Turkey; 3 Galen Research Center, Izmir Tinaztepe University, Buca, Izmir 35400 Turkey; 4 Department of Medical Biology and Genetics, Faculty of Medicine, Izmir Tinaztepe University, Buca, Izmir 35400 Turkey; 5 Dokuz Eylul University Hospital Medical Pharmacology Department, İzmir, Turkey; 6 Bolu Abant Izzet Baysal Üniversitesi Faculty of Medicine University Hospital Urology Department, Bolu, Turkey; 7 Edirne Sultan 1. Murat State Hospital, Edirne, Turkey; 8 Dokuz Eylul University Hospital Public Health Department, Izmir, Turkey

**Keywords:** Androgen receptor, Androgen receptor splice variant 7, Bladder cancer, Western blotting

## Abstract

**Purpose:**

The contribution of androgen receptors (AR) on bladder cancer has been demonstrated in pre-clinical studies, however in clinical studies, only the canonical AR (AR-FL) protein was measured by immunohistochemistry and conflicting results were obtained. To get better insight into the alterations of AR signalling, we used western blotting (WB) method and simultaneously measured both mRNA and protein levels of AR-FL and AR-V7.

**Methods:**

23 naive non-muscle invasive bladder cancer patients and 12 healthy individuals were included. AR-FL protein, AR-FL mRNA, AR-V7 protein and AR-V7 mRNA levels were quantitatively measured by WB and qRT-PCR.

**Results:**

While AR-FL protein and AR-V7 mRNA were significantly higher in bladder cancer, AR-FL mRNA and AR-V7 protein were lower. AR-V7 mRNA level was higher in patients with tumour size over 3 cm and AR-FL protein was higher in single tumours (*p* < 0,005). The small sampling size and the inclusion of only male participants were the main limitations.

**Conclusions:**

The increase of AR-FL protein in bladder cancer supports the contribution of the AR pathway in bladder cancer. The presence of high AR-FL protein despite low mRNA levels may be due to a disruption in post-transcriptional regulatory mechanisms. AR-V7 was demonstrated for the first time in bladder tissue and found significantly different in bladder cancer tissues. Our study reached new and valuable findings and will shed light on the studies that aim to clarify the role of the AR pathway in bladder cancer.

## Introduction

Bladder cancer is a frequent malignancy that is more prevalent in men [1]. In previous decades male dominant risk factors like tobacco usage and industrial exposure were held accountable for the gender difference. However, recent studies revealed these factors alone could not explain the male dominance. Even after adjustment of these factors, bladder cancer still has a 3 times higher incidence in men [2, 3]. To explain the male dominance, researchers hypothesised that androgen receptor (AR), may have a role in bladder cancer [1].

AR plays an important role in the development of male urogenital system. The role of AR is well-proved in organ malignancies such as prostate cancer, breast cancer, pancreas cancer and ovarian cancer [2]. Bladder cancer and AR relationship was also examined many times but the studies were conducted regarding only canonical, full-length AR (AR-FL) and AR splice variants (AR-Vs) were overlooked. There are more than 30 AR-Vs identified and AR-V7 is the most significant and well-characterized AR-V due to its proven role in prostate cancer, however, no studies have evaluated the association between AR-V7 and any other urogenital malignancy, including bladder cancer [3].

The contribution of AR-FL to bladder cancer has been well-proven in pre-clinical studies. Miyamoto et al. reported that exposure to N-butyl-N-(4-hydroxybutyl) nitrosamine (BBN) in mice resulted in bladder cancer in 92% of wild-type mice while 50% of castrated mice and none of AR-knocked out (ARKO) mice developed bladder cancer [4]. Clinical studies were based on AR protein measurement via immunohistochemistry (IHC) [1, 5–11]. Some studies reported increased AR-FL levels in bladder cancer samples, while other studies stated otherwise. Protein measurement with IHC is considered to be the main reason of this conflict [7]. Alternative protein measurement methods were suggested for clarification [7, 12]. Western blotting (WB) is a widely used method to obtain information about the quantity, molecular weight, and post-translational modifications of proteins. The WB method can detect much smaller amounts of proteins and can give quantitative results with high sensitivity and reproducibility compared to IHC while IHC is better for detecting the location of proteins [13, 14].

This is the first study to measure AR-FL protein levels in bladder cancer patients with WB. To get better insight into AR pathway, AR-V7 protein levels, AR-FL and AR-V7 mRNA levels (using qRT-PCR) were measured simultaneously.

The quantitative levels of AR-FL protein, AR-V7 protein, AR-FL mRNA and AR-V7 mRNA were analysed regarding clinicopathological features of bladder cancer and compared between bladder cancer tissues and healthy controls.

## Method

23 naive non-muscle invasive male bladder cancer (NMIBC) patients and 12 healthy males were prospectively included. Bladder tumour tissues were obtained with cold cup biopsy during primary transurethral resection. Healthy bladder tissues were obtained with cold cup biopsy during cystoscopy from healthy individuals with other cystoscopy indications (suspicion of bladder cancer, microscopic haematuria, lower urinary tract symptoms, etc.). PSA measurement, digital rectal examination and abdominal imaging were performed to exclude other urogenital malignancies. No participant had a history of other organ malignancy or anti-androgen treatment.

The levels of AR-FL protein, AR-V7 protein, AR-FL mRNA and AR-V7 mRNA were analysed and compared between two groups.

### Quantitative Polymerase-Chain Reaction (qPCR) analysis of AR-FL and AR-V7

Tissues were transferred immediately to Ribosave (Bio-Speedy, BS-NA-203-250), snap-frozen in liquid nitrogen, and stored at -20 °C. Total RNA isolation was performed from tissue samples using RNeasy Mini kit (Qiagen 74,104). RNA concentration and purity were controlled by NanoDrop 1000 spectrophotometer following isolation (Thermo, US). 500 nanograms of RNA per sample were used for cDNA synthesis with the RevertAid First Strand cDNA Synthesis kit (Thermo, K1622). cDNA synthesis was carried out in an Applied Biosystems, SimpliAmp Thermal Cycler. For AR-FL or AR-V7 primer evaluation, 10 or 20 ng of cDNA of each sample was applied per PCR, respectively. The PCR reaction conditions for primers were modified from the original Antonarakis et al. Publication by the Hohns Hopkins Group [15]. PCR primer pairs used for PCR targeted AR-FL (fw- CAGCCTATTGCGAGAGAGCTG, rev-GAAAGGATCTTGGGCACTTGC, [15]), AR-V7 (fw-CCATCTTGTCGTCTTCGGAAATGTTA, rev-TTTGAATGAGGCAAGTCAGCCTTTCT, [16]), and GAPDH (fw-GAAGGTGAAGGTCGGAGTC, rev-GAAGATGGTGATGGGATTTC). qPCR was performed using SYBR-Green fluorescent dye (Ampliqon, A323406) in an Applied Biosystems 7500 Fast Real-Time PCR Detection System. Samples were applied in quadruplicates. Relative gene expression of AR-FL and AR-V7 was normalized to GAPDH using the 2 -deltaCT method.

### Western blot analyses of AR-FL and AR-V7

**Fig. 1:**
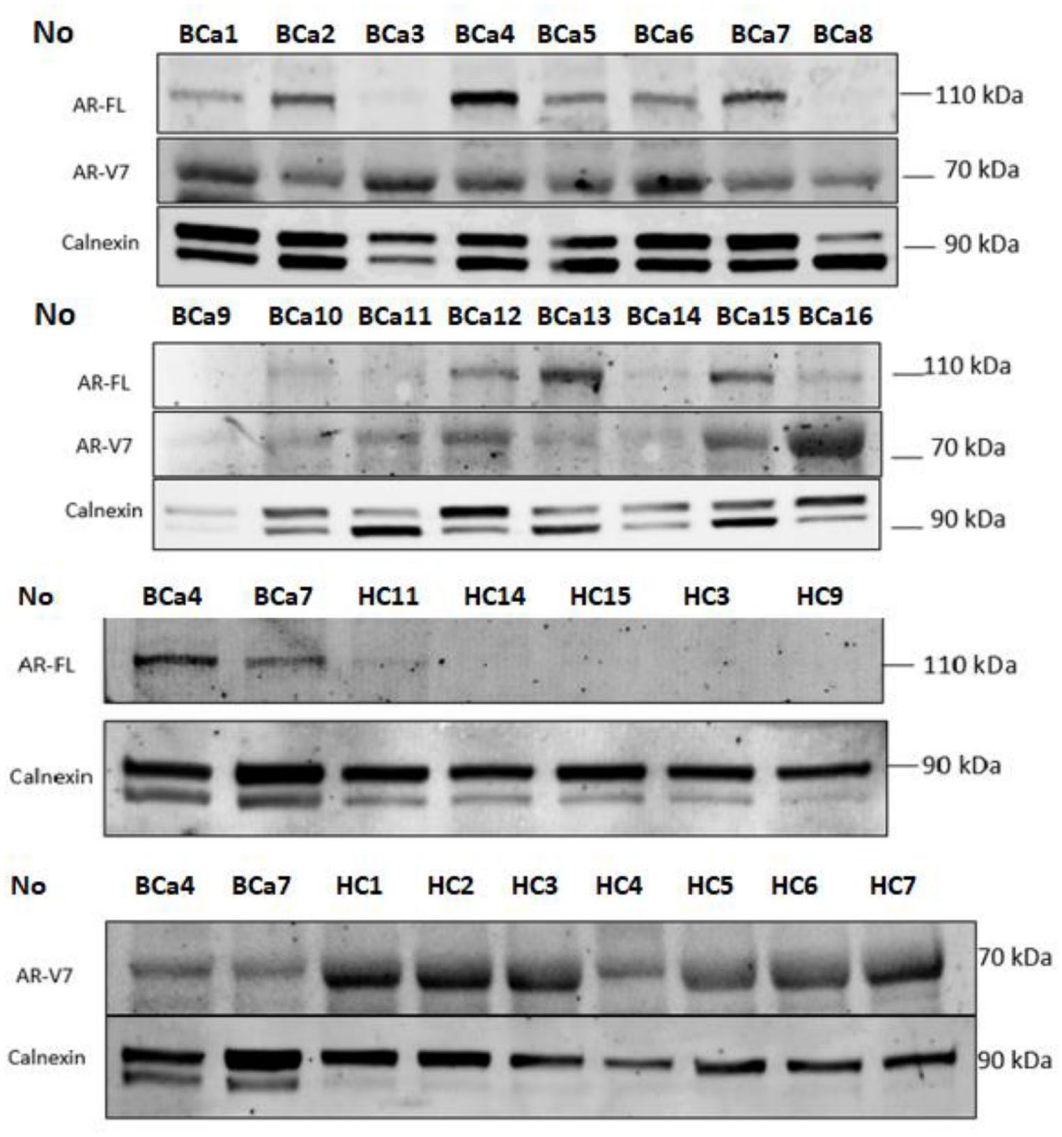
Western blotting and densitometric images of protein measurements of participants. BCa = Bladder Cancer, HC = Healthy Controls, AR-FL = Androgen Receptor Full Length, AR-V7 = Androgen Receptor Splice Variant 7

Tissues were snap-frozen in liquid nitrogen, and stored at -85° C. Tissue samples were homogenized in an ice-cold modified RIPA buffer containing a complete ultra mini protease inhibitor cocktail (Roche 05892970001) and phosSTOP (Roche) using pestles (Tmomas Scientific, 1226C62) as described before [17]. The homogenate was centrifuged at 15.000 x g for 20 min at + 4 °C. Protein lysates were prepared and analysed as described before [17] using 80 micrograms of protein. Blots were incubated with the following primary antibodies at indicated dilutions: Mouse anti-AR-FL (sc- 7305), 1:200, Mouse anti-AR-V7(Precision Antibody, AG10008) 1:500, rabbit calnexin (sc-11,397), 1:5000. Proteins were detected using fluorescence conjugated secondary anti-mouse (Licor 800CW: IRD 926-322-10) or anti-rabbit (Licor 680 RD:926-68071) antibodies both at 1:15000 and Chemidoc MP Imaging System (Biorad). Equal loading and transfer were confirmed by repeat probing for Calnexin. The band intensities were quantified as pixels by using ImageJ software (NIH) and calnexin measurements were used for internal calibration. 23 bladder cancer tissue protein levels were analysed with WB simultaneously. After that 12 healthy controls’ protein measurements were performed and bladder cancer tissues were re-measured in each gel for calibration (Fig. 1).

All statistical analyses were performed with the SPSS version 24.

## Results

Age and body mass index were similar but smoking was higher in the bladder cancer group (95,7% vs. 41,7%, *p* = 0,01, Table 1). Bladder tumour pathology was T1 in 8 (35%) patients and Ta in 15 (65%) patients. All T1 tumours and 2 Ta grade tumours were high grade (43%) and only two patients had carcinoma in situ (9%). According to the EORTC risk group, 7 patients (30%) were stratified as low-risk NMIBC, while 5 (22%) were medium-risk, 9 (39%) were high-risk and 2 (9%) were very high-risk NMIBC.

**Table 1:** Comparison of demographic characteristics and AR parameters between two groups

	Bladder Cancer (*n* = 23)	Healthy Controls (*n* = 12)	*p*
Age [median (min-max)] ^1^	67 (32–80)	72,5 (62–79)	0,11
Smoking History (%) ^2^	95,70	41,7	***0***,01*
Body Mass Index [median (min-max)] ^1^	28,4 (22,8–38,5)	27,2 (23,3–34)	0,96
AR-FL mRNA positivity, n (%)^2^	4%	92%	**< 0**,001*
AR-V7 mRNA positivity, n (%)^2^	83%	17%	**< 0**,001*
AR-FL Protein positivity, n (%)^2^	65%	0%	**0**,01*
AR-V7 Protein positivity, n (%)^2^	13%	83%	**< 0**,001*

*AR-FL* = Androgen Receptor Full Length, *AR-V7* = Androgen Receptor Splice Variant 7, *P* = Statistical Value

*Statistically significant difference, 1 = Student-t test was used in the analysis, 2 = Chi-square test was used in the analysis

### qRT-PCR and mRNA analysis

AR-FL and AR-V7 mRNA levels were measured by qRT-PCR method. Cut-off values were obtained by ROC analysis, values below were accepted as AR-FL or AR-V7 mRNA (-) and values above were accepted as (+).

**Fig. 2:**
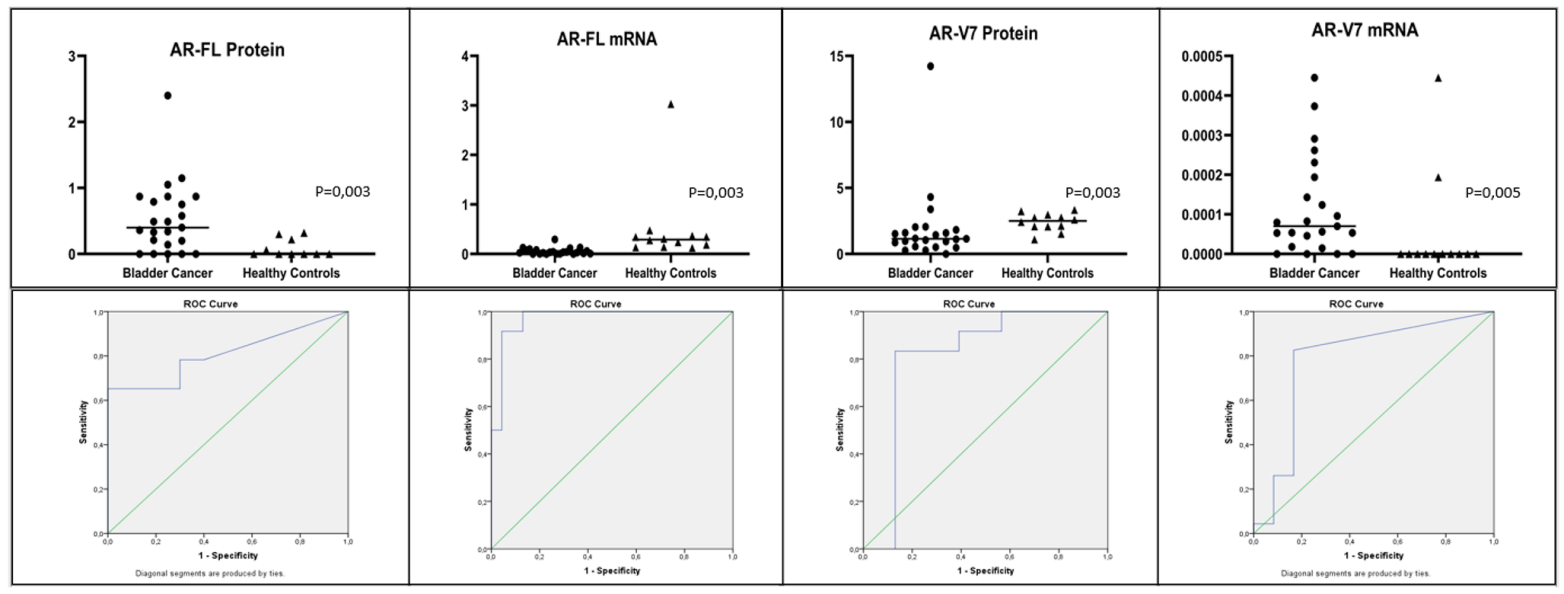
Comparison of AR parameters between two groups. AR-FL = Androgen Receptor Full Length, AR-V7 = Androgen Receptor Splice Variant 7, Mann-Whitney U test was used in the analysis for all parameters

AR-FL mRNA expression was higher in the control group (0,034 vs. 0.29 *p* = 0,003, Table 1; Fig. 2a). However, AR-V7 mRNA expression was higher in the bladder cancer group (0,0007 vs. 0,0000077; *p* = 0,005, Fig. 2b).

AR-FL mRNA positivity was %4 in bladder cancer group and 92% in control group while AR-V7 mRNA positivity was 17% in bladder cancer group and %83 in control group (*p* = 0,03 and < 0,001 respectively) (Table 1).

### Western blotting and protein analysis

AR-FL and AR-V7 protein levels were analysed by WB method and calnexin protein was used as the internal control (Fig. 1). Cut-off values were obtained by ROC analysis, values below were accepted as AR-FL or AR-V7 protein (-) and values above were accepted as (+).

AR-FL protein expression was higher in the bladder cancer group (0,4 vs. 0,06; *p* = 0,003, Fig. 2c; Table 1). AR-FL protein couldn’t be evaluated in two healthy individuals because no suitable quality samples were obtained for AR-FL protein expression. AR-V7 protein expression was higher in the control group (1,15 vs. 2,5 *p* = 0,003, Fig. 2d).

AR-FL protein positivity was 65% in bladder cancer group and 0% in control group. On the contrary AR-V7 protein positivity was 13% in bladder cancer group and %83 in control group (*p* = 0,01 and < 0,001 respectively) (Table 1).

AR-FL/AR-V7 protein ratio was higher in the bladder cancer group (0,38 vs. 0,02, *p* < 0,001). while AR-FL/AR-V7 mRNA ratio was lower (401,2 vs. 33536,7, *p* < 0,001).

No correlation was found between age, obesity, presence of CIS, EORTC risk group, tumour stage, tumour grade and any AR parameter. AR-V7 mRNA level was higher in patients with tumour size over 3 cm (*p* = 0,027, Table 2). AR-FL protein was higher in single tumours (*p* = 0,020, Table 2).

**Table 2:** Relations between AR parameters and tumour characteristics

	AR-FL Protein [median (min-max)]	AR-FL mRNA [median (min-max)]	AR-V7 Protein [median (min-max)]	AR-V7 mRNA [median (min-max)] *1000
Tumour Size^1^	*P* = 0,175	*P* = 0,055	*P* = 0,624	*P* = 0,027*
< 3 cm [7]	0,13 (0,06 − 0,87)	0,0048 (0,0013 − 0,09)	0,95 (0,20 − 3,39)	0,046 (0,007 − 0,08)
> 3 cm [16]	0,49 (0,06 − 2,4)	0,0425 (0,0006 − 0,29)	1,16 (0,27 − 14.,23)	0,109 (0,007 − 0,44)
Number of Tumours^1^	*P* = 0,020*	*P* = 0,319	*P* = 0,919	*P* = 0,101
Single [6]	0,80 (0,36 − 2,4)	0,0612 (0,0007 − 0,29)	1,13 (0,57 − 1,83)	0,168 (0,01 − 0,44)
Multiple [17]	0,32 (0,06 − 1,05)	0,0298 (0,0006 − 0,13)	1,17 (0,2-14.23)	0,053 (0,007 − 0,37)
Stage^1^	*P* = 0,548	*P* = 0,825	*P* = 0,190	*P* = 0,875
Ta [15]	0,40 (0,06 − 2,4)	0,029 (0,0007 − 0,29)	0,97 (0,2–4,32)	0,056 (0,007 − 0,44)
T1 [8]	0,34 (0,06 − 1,05)	0,036 (0,0006 − 0,12)	1,38 (0,27 − 14,23)	0,075 (0,007 − 0,37)
Grade^1^	*P* = 0.232	*P* = 0,186	*P* = 0,446	*p* = 0,225
Low-Grade [13]	0.49 (0.06–2.4)	0,0617 (0,0013 − 0,29)	0,97 (0,2–4,32)	0,082 (0,007 − 0,44)
High-Grade [10]	0.28 (0.06–1.05)	0,0302 (0,0006 − 0,12)	1,16 (0,27-14.23)	0,058 (0,007 − 0,37)
Presence of Cis^1^	*P* = 0,332	*P* = 0,126	*P* = 0,443	*P* = 0,237
No [21]	0,49 (0,06 − 2,4)	0,0376 (0,0006 − 0,29)	1,15 (0,20 − 14,23)	0,079 (0,007 − 0,44)
Yes [2]	0,20 (0,06 − 0,36)	0,0042 (0,0007 − 0,007)	2,25 (1,12 − 3,39)	0,030 (0,010 − 0,040)
EAU Risk Group^2^	*P* = 0,405	*P* = 0,587	*P* = 0,446	*P* = 0,232
Low [7]	0,82 (0,06 − 2,40)	0,078 (0,0013 − 0,29)	1,11 (0,2–2,05)	0,134 (0,007 − 0,445)
Intermediate [5]	0,45 (0,20 − 0,75)	0,066 (0,0145-0,13)	1,72 (0,28 − 4,32)	0,155 (0,053 − 0,262)
High [9]	0,42 (0,06 − 1,05)	0,038 (0,0006 − 0,13)	2,49 (0,27 − 14,23)	0,093 (0,007 − 0,372)
Very-High [2]	0,27 (0,06 − 0,49)	0,021 (0,0076 − 0,03)	2,28 (1,18 − 3,39)	0,063 (0,046 − 0,080)

*AR-FL* = Androgen Receptor Full Length, *AR-V7* = Androgen Receptor Splice Variant 7, *Cis* = carcinoma in situ, *EORTC* = The European Organisation for Research and Treatment of Cancer, *P* = Statistical Value

*Statistical difference, 1 = Mann-Whitney U test was used in the analysis, 2 = One way ANOVA test was used in the analysis

## Discussion

The bladder epithelium is derived from the urogenital sinus endoderm, and AR-FL signalling plays a role in the development of this tissue. The AR pathway has been demonstrated to play a role in several organ malignancies [18, 19]. Several studies compared AR-FL protein levels between the bladder cancer and healthy tissues and the results are controversial. Some authors reported higher levels of AR-FL protein in bladder cancer [8–10] while others reported the opposite [1, 5, 8–10, 20]. AR-FL protein was measured with IHC in all studies and AR-FL protein positivity rates were observed to vary widely between 8.75% and 75% [7]. The high variation of positivity rate between studies raised doubts about the reliability of IHC. Alternative protein measurement methods such as WB were suggested to overcome this problem [7]. There are several advantages of WB method including reproducibility, having specific epitopes, detection of even low amounts of protein, and high quantitative sensitivity while IHC has a higher threshold for detection and is an observer-dependent method [13, 14]. Our study is the first to measure AR-FL protein levels by WB. According to our results AR-FL protein is increased in bladder cancer.

Triphati et al. stated that simultaneous measurement of mRNA and protein levels is needed to resolve this conflict and to better understand the importance of AR signalling in bladder cancer [12]. Our study is the first to examine AR-FL mRNA and AR-FL protein levels simultaneously in bladder cancer. mRNA expression is one of the first major steps of protein synthesis and high AR-FL protein is known to suppress AR-FL mRNA expression by feedback [21, 22]. In our study, AR-FL mRNA levels were lower in bladder cancer. We think that the decrease in mRNA is due to the suppression by negative feedback rather than a contributing mechanism to bladder cancer. We also believe that the main role player for bladder cancer development is increased AR-FL protein in this pathway. The mRNA-independent increase of AR-FL protein may be due to a dysregulation that occurs in a step after mRNA transcription, such as translation, splicing, miRNA, protein degradation, folding or degradation.

There is only one study evaluating the association between bladder cancer and AR-V’s [23]. Katleba et al. detected AR-V19 expression was present in bladder cancer and stated that treatments targeting AR pathway may be effective in bladder cancer [23]. It was also shown that castration prevented cancer in half of the mice, while completely knocking-out the androgen receptor prevented cancer in all mice [4]. This finding suggests that androgen-independent activation of AR plays a role in development of bladder cancer and AR-V7 pathway is the most well-known example of androgen independent activation of AR [24].

Our study is the first to analyse AR-V7 mRNA and protein levels in bladder cancer along with AR-FL, and even the first one to show the presence of AR-V7 in bladder tissue. According to our findings, AR-FL protein becomes the dominant protein and the amount of AR-V7 protein decreases in bladder cancer, whereas AR-V7 mRNA level increases. However, it is very difficult to discuss our findings regarding AR-V7 and bladder cancer, the underlying mechanism of AR-FL protein increase or loss of regulation may also simultaneously impede splicing and/or other AR-V7 synthesis steps. The reduction of the end product AR-V7 protein may eliminate the suppressive effect on AR-V7 mRNA and increase in AR-V7 mRNA expression. Clarifying the step in these complex pathways that dysregulation occurs and causes changes in the four mentioned AR parameters is a research topic for future studies.

In our study, we were able to measure AR-FL or AR-V7 protein levels semi-quantitatively in all patients by WB. This demonstrates the superiority of WB and we think that usage of WB may be optimal for AR protein measurements.

The main limitations of our study are the small sampling size and the inclusion of only male participants. A possible relationship between AR parameters and clinicopathological features of the tumour may have been overlooked due to the small sample size. Inclusion of only NMIBC patients is another limitation, several studies have demonstrated lower AR-FL levels in MIBC due to poorer differentiation [1, 8]. We aimed to keep our patient group as homogenous as possible since we were limited by the sample size.

One of the strengths of our study is that only naive NMIBC patients who had not received any treatment were included, as we know that treatments can alter the genetics/molecular patterns of cancer. Another strength is that tissues were obtained fresh immediately after biopsy, not from paraffin blocks, and were stored under appropriate conditions until evaluation.

The AR pathway plays an important role in bladder cancer. Our outcomes obtained by a novel approach at both mRNA and protein levels of AR-FL and AR-V7 will shed light on the studies that will clarify the mechanism of AR pathway in bladder cancer development. mRNA stability, micro-RNA related pathways, post-transcriptional splicing, translation, post translational modifications and protein degradation may be the dysregulated steps in AR mRNA-protein pathway that led to our findings. Comparative transcriptomic and proteomic analysis will provide insight into key genes involved in these regulatory steps. Better understanding of these steps will provide evidence for the use of AR-targeted therapies in the treatment of bladder cancer.

## Data Availability

Data is availiable for this study.
